# Draft Genome Sequence of *Bacillus atrophaeus* UCMB-5137, a Plant Growth-Promoting Rhizobacterium

**DOI:** 10.1128/genomeA.00233-13

**Published:** 2013-06-20

**Authors:** Wai Yin Chan, Kristin Dietel, Svitlana V. Lapa, Lilija V. Avdeeva, Rainer Borriss, Oleg N. Reva

**Affiliations:** Department of Biochemistry, Bioinformatics and Computational Biology Unit, University of Pretoria, Hillcrest, Pretoria, South Africaa; Department of Microbiology and Plant Pathology, University of Pretoria, Hillcrest, Pretoria, South Africab; ABiTEP CmbH, Berlin, Germanyc; Department of Antibiotics, Institute of Microbiology and Virology NAS of Ukraine, Kiev, Ukrained

## Abstract

*Bacillus atrophaeus* UCMB-5137 shows an extraordinary activity in root colonization and plant and crop protection. Its draft genome sequence comprises 21 contigs of 4.11 Mb, harboring 4,167 coding sequences (CDS). The genome carries several genes encoding antimicrobial lipopeptides and polyketides. Multiple horizontally acquired genes of possible importance for plant colonization were also found.

## GENOME ANNOUNCEMENT

With the increasing demand for ecologically safe biotechnological pesticides in the plant and crop industry, which may be alternatives to chemical pesticides, endophytic and rhizospheric plant growth-promoting *Bacillus* strains are widely used in modern biotechnology (1). A vast majority of biotechnological strains belong to *Bacillus amyloliquefaciens* subsp. *plantarum* (2, 3, 4). As a phylogenetically distinct species, *Bacillus atrophaeus* strain UCMB-5137 also has the ability to colonize plant roots and to inhibit the growth of fungal and bacterial phytopathogens on inoculated seedlings and harvested fruits (5). It was isolated from grass rhizosphere in Ukraine in 1976 and formulated as an active component of the biopesticide Fruktophit, which is particularly used on soft berries (strawberries and grapes) and root vegetables in storage facilities. Compared to other environmental plant-associated bacterial genomes, the genome sequence of *B. atrophaeus* UCMB-5137 may provide insight into the evolutionary adaptation to its niche and the discovery of putative antimicrobial compounds.

The genome was sequenced by Macrogen Inc. (Republic of Korea), using Illumina HiSeq 2000 technology. Quality trimmed DNA reads conferring an average of 350-fold coverage were *de novo* assembled by Velvet 1.2.03 and CLC Genomics Workbench 5. The draft assembled contigs were aligned against the complete reference genome sequence of *B. atrophaeus* 1942 using the r2cat program (6) and the NG Aligner tool of the NCBI Genome Workbench v.2.5.5. The overlapping fragments were merged into larger contigs. Open reading frame (ORF) prediction and functional annotation of the predicted genes were done by GeneMark.hmm (7), RAST (8), and BLASTp against the known protein sequences of 22 reference genomes of *B. atrophaeus*, *B. amyloliquefaciens*, and *Bacillus subtilis*, the sequences of which are available from NCBI and PATRIC (9) databases. The draft assembly consists of 21 contigs with 4,114,051 bp and a G+C content of 43.37%. A total of 4,167 putative coding sequences (CDS) were identified, of which 3,025 are orthologous genes present in all 22 reference genomes.

Six giant gene clusters of nonribosomal peptide-synthetase (NRPS)/polyketide synthase (PKS) genes were found, among them, those for surfactin, bacillibactin, bacitracin, mycosubtilin, plipastatin/fengycin, and bacillaene operons, which are commonly associated with other sequenced *B. subtilis* and *B. amyloliquefaciens* genomes. Genome comparison revealed sixty unique genes in *B. atrophaeus* UCMB-5137, and most of them were localized in horizontally acquired genomic islands. These regions comprised many phage-associated genes, which may indicate that phages and the horizontal gene exchange in general played an important role in the evolution of plant-associated *Bacillus* species. Sixteen genes shared by UCMB-5137 and other plant-associated *B. amyloliquefaciens* subsp. *plantarum* strains and *B. subtilis* BSn5 were absent in other *B. atrophaeus* strains. These genes might be responsible for plant colonization, together with an operon containing LysM peptidoglycan-binding proteins that are possibly involved with biofilm formation (10).

### Nucleotide sequence accession number.

The whole-genome sequence of *B. atrophaeus* UCMB-5137 has been deposited in NCBI with the accession no. APIW00000000.
